# Dynamic Neural Processing of Linguistic Cues Related to Death

**DOI:** 10.1371/journal.pone.0067905

**Published:** 2013-06-28

**Authors:** Xi Liu, Zhenhao Shi, Yina Ma, Jungang Qin, Shihui Han

**Affiliations:** Department of Psychology, PKU-IDG/McGovern Institute for Brain Research, Peking University, Beijing, P. R. China; University of Pennsylvania, United States of America

## Abstract

Behavioral studies suggest that humans evolve the capacity to cope with anxiety induced by the awareness of death’s inevitability. However, the neurocognitive processes that underlie online death-related thoughts remain unclear. Our recent functional MRI study found that the processing of linguistic cues related to death was characterized by decreased neural activity in human insular cortex. The current study further investigated the time course of neural processing of death-related linguistic cues. We recorded event-related potentials (ERP) to death-related, life-related, negative-valence, and neutral-valence words in a modified Stroop task that required color naming of words. We found that the amplitude of an early frontal/central negativity at 84–120 ms (N1) decreased to death-related words but increased to life-related words relative to neutral-valence words. The N1 effect associated with death-related and life-related words was correlated respectively with individuals’ pessimistic and optimistic attitudes toward life. Death-related words also increased the amplitude of a frontal/central positivity at 124–300 ms (P2) and of a frontal/central positivity at 300–500 ms (P3). However, the P2 and P3 modulations were observed for both death-related and negative-valence words but not for life-related words. The ERP results suggest an early inverse coding of linguistic cues related to life and death, which is followed by negative emotional responses to death-related information.

## Introduction

Humans have the desire for continued existence in spite of consciously knowing death’s inevitability. The existential concern constitutes one of the basic motivations of human life and influences our behaviors and attitudes significantly [1], [2]. Mortality salience leads to positive attitudes toward people and ideas that support their worldview [3] and results in endorsement of positive personality descriptions or increased self-esteem [4], [5]. There has been increasing evidence of behavioral studies [6], [7] that support the Terror Management Theory [8], which proposes that reminders of death cause sustained anxiety and that humans mitigate such anxiety through the development and maintenance of a dual-component anxiety buffer including cultural worldview and self-esteem.

While previous behavioral studies suggest a mechanism to cope with the anxiety associated with existential concern, little is known about neurocognitive processes underlying online death-related thoughts that initiate the anxiety and related coping processes. Early studies suggest that emotional processing may be a component of death-related thoughts. For example, by recording facial electromyography (EMG) to masked presentations of either the word “dead” or “pain”, Arndt et al. [9] observed greater EMG specifically during exposure to subliminal death but not subliminal pain primes from the corrugator muscle that is involved in emotional facial expression. DeWall and Baumeister [10] found that human individuals showed increased accessibility of positive emotional information and assigned more weight to positive emotion during judgments of word similarity after contemplating death than after contemplating dental pain. While these findings suggest the involvement of emotional processing in death-related thoughts, the exact underlying neural mechanisms remain unclear.

Functional magnetic resonance imaging (fMRI) has recently been used to investigate the neural correlates of death-related thoughts in humans. Han et al. [11] first scanned human adults during a modified Stroop task that required color naming of death-related, negative-valence, and neutral-valence words. It was found that both death-related and negative-valence words increased hemodynamic responses in brain areas associated with emotional arousal and regulation (e.g., the precuneus/posterior cingulate and lateral frontal cortex). Another fMRI study also reported increased activity in the amygdala and the ventral anterior cingulate cortex to items related to fear of death compared to those related to dental pain [12]. While these fMRI results suggest the involvement of emotional responses in death-related thoughts, Han et al. [11] found a unique process of death-related words that was characterized by decreased activity in the insula that mediates representations of the sentient self [13]. Recently, Shi and Han [14] further showed that the unique neural process of death-related words (e.g., decreased insular activity) was evident in the sustained neural activity related to a death-relevance judgment task but not in the transient neural activity engaged in trial-specific processes of death-related linguistic cues. These findings suggest that, on the one hand, emotion-related neural processes are involved in death-related thoughts. On the other hand, specific neural mechanisms may be engaged in the processing of linguistic cues related to death (e.g., decreased insular activity). However, the fMRI results failed to uncover the time course of neural processing of death-related linguistic cues due to the low time resolution of blood oxygen level dependent signals.

Given the significance of death-related signals for life, humans may have evolved another mechanism, in addition to emotional responses to death-related information, for early processing of death-related signals. In addition, according to Terror Management Theory [1], [8], the human mind contains mechanisms that generally keep thoughts of death from becoming conscious and remove such thoughts from focal attention when they do. Such mechanisms require early detection of death-related information that induces thoughts of death and negative emotional responses. Therefore, an early process may be engaged in detection of death-related cues prior to emotional responses to death-related information. Moreover, recent research found that manipulating the value of human life subsequently increased death-related-thought accessibility [15], which, according to the authors, reflects the fact that death represents scarcity of life. Thus, it may be further hypothesized that detection of death-related and life-related cues may take place at a similar early stage but engage reverse neural modulations to encode the opposite aspects of life.

The current study tested these hypotheses by recording event related potentials (ERPs) from young adults who were presented with death-related, life-related, negative-valence, and neutral-valence words, as shown in Tables 1–5. We chose linguistic cues related to death and life because they are unique for humans, are not contaminated by complicated visual and emotional cues, and are comparable in low-level sensory-perceptual processing. In a modified Stroop task participants were asked to identify colors of these words in order to control for the depth of semantic processing of different types of words. Previous studies using the emotional Stroop task have shown that, relative to neutral-valence words, negative-valence words elicited positive-going ERPs as early as 250 ms after stimulus onset over the parietal area [16] and induced modulations of the occipital activity (e.g., P1 [17] and N1 [18]. Naming colors of threat words also resulted in early modulations of a frontal positivity at 150–250 ms (P2) [19], [20]. However, as only a small proportion of threat words were related to death in the previous studies, it is unclear whether the P2 effect is specific to the processing of linguistic cues of death or reflects general processing of negative-valence of threat words. Other ERP studies have shown that a frontal/central N1 was modulated by categorization of images in terms of lifehood (i.e., animals versus man-made objects). Explicit categorization of animals versus artifacts decreased the N1 amplitudes to images of animals [21], whereas implicit categorization of animals versus artifacts increased the N1 amplitudes to images of animals [22]. However, as death-related stimuli were not included in these studies, it remains unclear if death-related information can be coded at this early stage of processing.

**Table 1:** Death-related words (Chinese and English translation) used in Experiment 1.

Nouns				Verbs			
??	funeral music	??	hanging	??	attend a funeral	??	lose one’s life
??	cancer	??	air crash	??	hold a funeral procession	??	suffer death
??	debris	??	skull	??	execute	??	visit a grave
??	tragedy	??	coffin	??	assassinate	??	slaughter
??	landmine	??	mourning hall	??	mourn	??	commit homicide
??	poison gas	??	mausoleum	??	murder by poisoning	??	decapitate
??	poison	??	graveyard	??	ingest poison	??	hang oneself
??	cemetery	??	murder	??	cremate	??	visit a grave
??	grave	??	corpse	??	bury	??	pass away
??	cremains	??	murderous weapon	??	perish	??	massacre
??	shipwreck	??	murderer	??	plot to murder	??	assault
??	funeral wreath	??	Hades	??	murder	??	die young
??	underworld	??	corpse	??	drown	??	suffocate
??	death anniversary	??	hell	??	execute by shooting	??	suicide
??	altar	??	funeral	??	pass away	??	cut one’s own throat

**Table 2:** Neutral-valence words (Chinese and English translation) used in Experiments 1 and 3.

Nouns				Verbs			
??	shuttle bus	??	auditorium	??	chat	??	irrigate
??	cup	??	pocket change	??	pump water	??	pay tax
??	industry	??	corridor	??	recompense	??	blossom
??	the Yangtze River	??	tire	??	inquire	??	mind the house
??	shirt	??	model	??	type words	??	take a photograph
??	moral principles	??	charcoal	??	play instruments	??	knock at the door
??	cabaret	??	butter	??	order TV program	??	apply for a job
??	Forbidden City	??	hill	??	take (medicine, etc.)	??	negotiate
??	pot cover	??	annual income	??	close a door	??	eliminate illiteracy
??	sweet potato	??	timber	??	plant a tree	??	turn off the motor
??	flower	??	candy	??	gather	??	wash feet
??	honored guest	??	swan	??	refuel	??	write
??	handout	??	toy	??	discount	??	repair a road
??	lottery	??	carton	??	overhaul	??	perform
??	test score	??	pork	??	give a lecture	??	play the finger-guessing game

**Table 3:** Life –related words (Chinese and English translation) used in Experiment 2.

Animals				Plants			
??	turtle	??	donkey	??	pinus	??	elm
??	lion	??	spider	??	willow	??	narcissus
??	leopard	??	penguin	??	palm	??	chrysanthemum
??	bear	??	flea	??	jujube tree	??	lotus
??	mouse	??	locust	??	herbs	??	lotus root
??	frog	??	hippo	??	hibiscus	??	orchid
??	hawk	??	ribbonfish	??	crape myrtle	??	asparagus
??	monkey	??	pigeon	??	cypress	??	peony
??	crab	??	carp	??	holly	??	China rose
??	dragonfly	??	lizard	??	Sophora japonica	??	rose
??	wasp	??	swan	??	willow	??	strawberry
??	elephant	??	magpie	??	pear tree	??	plum flower
??	bee	??	cuckoo	??	poplar	??	ginkgo
??	elk	??	seagull	??	metasequoia	??	white birch
??	buffalo	??	migratory bird	??	boxwood	??	camellia

**Table 4:** Life-unrelated words (Chinese and English translation) used in Experiment 2.

Nouns							
??	wineglass	??	sandals	??	jersey	??	bath towel
??	kitchenknife	??	leatherboots	??	shirt	??	soap
??	mirror	??	slippers	??	bras	??	bathtub
??	basket	??	trousers	??	dress	??	voltage tester
??	wok	??	ring	??	teamuniform	??	table lamp
??	electricmeter	??	necklace	??	suit	??	bookcase
??	cup	??	boat shoes	??	underpants	??	wooden bench
??	spoon	??	Leathershoes	??	shorts	??	wall lamp
??	teacup	??	raincoat	??	sweater	??	swivel chair
??	square table	??	earring	??	straw hat	??	bed sheet
??	deck chair	??	belt	??	scarf	??	bookshelf
??	fan	??	watch	??	sunglasses	??	drawer
??	umbrella	??	glasses	??	trousers	??	tea table
??	carton	??	pajamas	??	cloth shoes	??	square stool
??	towel	??	tie	??	schooluniform	??	bench

**Table 5:** Negative-valance words (Chinese and English translation) used in Experiment 3.

Nouns				Verbs			
??	incriminatingevidence	??	malformation	??	hinder	??	fabricate
??	idiot	??	quarrel	??	cover up	??	be biased
??	wastrel	??	sulk	??	discarded	??	swindle
??	malpractice	??	fallacy	??	plagiarize	??	infringe
??	miser	??	tough obstacle	??	ridicule	??	disturb
??	prejudice	??	adversecircumstance	??	flaunt	??	harass
??	shame	??	coward	??	boast	??	instigate
??	fool	??	traitor	??	sabotage	??	renege
??	sluggishness	??	scam	??	spite	??	tell a lie
??	vices	??	cheater	??	envy	??	instigate
??	annoyance	??	a road ofevil	??	degenerate	??	nitpick
??	conspiracy	??	brute	??	slander	??	evade taxes
??	trick	??	defect	??	instigate	??	humiliate
??	negligence	??	lie	??	exaggerate	??	mislead
??	falsehood	??	trap	??	hoodwink	??	intimidate

A recent research recorded ERPs to death-related, death-unrelated unpleasant/pleasant, and neutral-valence words [23]. In Study 1 death-related and death-unrelated words were intermixed with pseudowords and participants were asked to perform word vs. pseudoword judgments. In Study 2 death-related and death-unrelated words were intermixed with target words that described animals and required behavioral responses. In both studies Klackl et al. [23] found that a larger amplitude of late positive potential at 400–800 ms for death-related relative to death-unrelated words, whereas earlier neural processes did not differentiate between death-related and death-unrelated words. Because the prior fMRI study has shown that the neural activity that characterized the processing of death-related words can be sustained for a few minutes [14], intermixed presentations of death-related and death-unrelated unpleasant/pleasant words may result in interactions between the neurocognitive processes of different types of linguistic cues (e.g., the effect of death-related words on brain activity is sustained and thus influences the neural activity to unpleasant words) and thus weaken the early ERP effect associated with the processing of death-related words.

The present study examined the dynamic neural process of linguistic cues of death by recording ERPs to death-related/neutral-valence words in Experiment 1, life-related/life-unrelated words in Experiment 2, and negative-valence/neutral-valence words in Experiment 3. In each experiment either death-related, life-related, or negative-valence words were compared with neutral-valence words that were death/life unrelated and of neutral valence in order to identify the neural activity specifically involved in the processing of lifehood and negative valence of words. Death-related, life-related, and negative-valence words were presented separately in three experiments in order to avoid the interaction between the neural processes of death-relevance, life-relevance, and word valence. Word frequency, length, and number of strokes of Chinese characters used in words were matched in all conditions to control for perceptual and low-level word processing. Participants were asked to identify colors of words in all experiments. The amplitudes of difference waves, which were obtained by subtracting ERPs to neutral-valence words from those to death-related, life-related, and negative-valence words, were compared across experiments to confirm the differential neurocognitive processes of different type of words. The sequence of the three experiments was counterbalanced across subjects in order to reduce any effects such as habituation. We predicted that the neural activity that differentiates between death-related and neutral-valence words may occur earlier than the neural activity differentiating between negative-valence and neutral-valence words. In addition, the neural activity that differentiates between life-related and life-unrelated words may occur as early as the neural activity differentiating between death-related and neutral-valence words, but in an opposite pattern.

Given that optimism and pessimism reflect positive and negative attitudes toward life that may be associated with death-related mental problem (e.g., depression) [24] and behaviors (e.g., suicide) [25], we asked participants to complete the Life Orientation Test (LOT) [26] which estimates individuals’ dispositional optimism and pessimism attitudes toward life. This was then used to assess whether neural modulations of death-related/life-related words could predict subjects’ attitude difference. We also employed two questionnaires, i.e. the Death Anxiety Scale [27] and the Existential Anxiety Questionnaire [28], to assess the association between anxiety traits related to death and neural modulations of death-related/life-related words. Given the sex differences in fear of death [29] and brain structures involved in emotional processing [30], the current study recruited only female subjects to avoid potential confounds of sex differences.

## Materials and Methods

### Participants

Twenty-five right-handed female university students (19–29 years of age, mean age = 23.0±2.4) participated in the present study as paid volunteers. All subjects reported normal color vision and normal or corrected-to-normal visual acuity, and no history of neurological or psychiatric problems. Informed written consent was obtained prior to the study. This study was approved by a local ethics committee at the Department of Psychology, Peking University. All participants were debriefed and explained the purpose of this research after the study. Handedness was assessed using the Edinburgh Handedness Inventory [31].

### Stimuli

Each word used in the current study consisted of two Chinese characters, as shown in Tables 1–5. Sixty death-related words (30 nouns and 30 verbs) and sixty neutral-valence words (30 nouns and 30 verbs) were used in Experiment 1. Word frequency was matched for different categories of words (death-related words: M±SD = 4.567±4.785; neutral-valence words: M±SD = 4.600±3.920; t(118) = −0.417, p = 0.967) [32]. Sixty life-related words and sixty life-unrelated words were used in Experiment 2. The word frequency (life-related words: M±SD = 4.483±4.257; life-unrelated words: M±SD = 4.467±4.838) was matched with those of death-related words and neutral-valence words used in Experiment 1 (F(3, 236) = 0.0125, p = 0.998). Sixty negative-valence words (30 nouns and 30 verbs) that were unrelated to death and sixty neutral-valence words were used. The word frequency of negative valence words (M±SD = 4.217±3.289) was matched with those of death-related words, neutral-valence words used in Experiment 1 (F(2, 177) = 0.165, p = 0.848), and life-related and life-unrelated words used in Experiment 2 (F(2, 177) = 0.0767, p = 0.926). The neutral-valence words used in Experiment 1 were also used in Experiment 3 for the purpose of comparison.

An independent group of 46 subjects were asked to rate death-related words, negative-valence words and neutral-valence words in terms of semantic relevance to death and emotional arousal. The results were reported in our previous work [11]. We also asked an independent group of 24 subjects to rate the stimuli in terms of life-relevance (“How is this word relevant to life?” 0 = not at all relevant, 10 = extremely relevant) and arousal (“How strong is your emotional response induced by this word?” 0 = no at all, 10 = extremely strong) on an 11-point Likert scale. Paired t-tests showed that life-relevance was rated significantly higher for life-related than life-unrelated words (7.72±1.95 vs. 2.55±2.24, t(23) = 10.89, p<0.001). Rating scores of arousal were low for both life-related and unrelated words though slightly higher for life-related than life-unrelated words (1.21±1.09 vs. 0.61±1.14, t(23) = 4.896, p<0.001).

### Procedure

In all experiments each word was presented in the center of a screen against a gray background during EEG recording. Each character subtended a visual angle of 2.2×3.0° (width×height) at a viewing distance of 90 cm. Half of the words in each category were colored blue and half orange with both colors matched in brightness. There were two blocks of 120 trials in each experiment while the electroencephalograph (EEG) was recorded. Each block consisted of 60 death-related words and 60 neutral-valence words in Experiment 1, 60 life-related words and 60 life-unrelated words in Experiment 2, 60 negative-valence words and 60 neutral-valence words in Experiment 3. Stimuli in each block were presented in a random order. Each word was presented for 400 ms followed by an inter-stimulus interval that varied randomly from 1000 to 1800 ms during which a fixation cross was presented at the center of the screen. In all experiments participants pressed one of the two buttons as accurately and quickly as possible to indicate the color of each word using the left or right index fingers. The correspondence between word colors and responding fingers was counterbalanced across subjects. The order of the three experiments was counterbalanced across participants.

After EEG recording sessions, participants rated all death-related words and neutral-valence words on an 11-point Likert scale on the degree of evoked negative emotional arousal (0 = not at all, 10 = extremely strong). Individuals’ dispositional optimism and pessimism related to levels of stress and vigilance towards negative emotional events were measured using the LOT [26], which contains 11 items on a 7-point Likert scale (0 = strongly disagree, 6 = strongly agree) with a 5-item optimism subscale (e.g., “I am always optimistic about my future”) and a 6-item pessimism subscale (e.g., “I always have bad luck”). The Death Anxiety Scale with 15 true-false items (DAS) [27] and Existential Anxiety Questionnaire with 13 true-false items (EAQ) [28] were administrated to measure individuals’ degree of anxiety about death. Both are scored 0 or 1 such that a high score indicates a high degree of death anxiety.

### EEG Recording and Analysis

The electroencephalogram (EEG) was continuously recorded using a Neuroscan system. EEG was recorded from 62 scalp Ag/AgCl electrodes mounted on an elastic cap according to the extended 10–20 system with the addition of two mastoid electrodes. The EEG recording system has a gain of 500 and a resolution of 0.168µV/LSB. The mean of the right and left mastoid electrodes was used as reference during online EEG recording. The impedance of each electrode was kept below 5 kΩ. Eye blinks and vertical eye movements were monitored with electrodes located above and below the left eye. The horizontal electro-oculogram was recorded from electrodes placed 1.5 cm lateral to the left and right external canthi. The EEG was amplified (band pass 0.01–100 Hz) and digitized at a sampling rate of 250 Hz. A band-pass filter of 0.01–40 Hz was applied during offline EEG processing using Scan 4.3. The ERPs in each condition were averaged separately offline with an epoch beginning at 200 ms before stimulus onset and continuing for 1200 ms. Trials contaminated by eye blinks, eye movements, or muscle potentials exceeding ±50 µV at any electrode were excluded from the average, resulting in rejection of about 12% trials from further data analysis. The baseline for ERP measurements was the mean voltage of a 200 ms prestimulus interval and the latency was measured relative to the stimulus onset.

Visual inspection of grand average ERPs identified a negativity at 84–120 ms (N1), followed by a positivity at 124–300 ms (P2) with the maximum amplitude over the frontal/central regions and a positivity at 300–500 ms (P3) with the maximum amplitude over the central/parietal regions. Stimuli also elicited a positivity at 92–132 ms over the occipital areas (P1), which was followed by a negativity at 150–200 ms over the lateral occipital electrodes (N170) and a positivity at 232–440 ms over the parietal/occipital electrodes (P3). The mean amplitudes of the N1, P2, and P3 waves were calculated and subjected to statistical analyses at frontal (F1–F6, FZ), fronto-central (FC1–FC6, FCZ) and central (C1–C6, CZ) electrodes. We also calculated and analyzed the mean amplitudes of the P1, N170 and P3 over the posterior electrodes (parietal: P3–P8; parieto-occipital: PO3–PO8; occipital: O1–O2). The mean ERP amplitudes were subjected to repeated-measure analyses of variance (ANOVAs) with Hemisphere (electrodes over the left or right hemisphere) and Death Relevance (death-related vs. neutral-valence words) in Experiment 1, Life Relevance (life-related vs. life-unrelated) in Experiment 2, and Valence (negative vs. neutral) in Experiment 3 as within-subjects variables at each pair of electrodes over the left and right hemisphere. Paired t-tests were conducted at each individual electrode along the midline cortical structure to examine the effect of Death Relevance in Experiment 1, Life Relevance in Experiment 2, and Valence in Experiment 3. Difference waves were obtained by subtracting ERPs to neutral-valence words from those to death-related words and the amplitudes of the difference waves were subjected to correlation analyses.

## Results

### Experiment 1

#### Behavioral performances

Reaction times (RTs) with correct responses within three standard deviations from the mean were included for analysis. Paired t-tests did not show significant differences between death-related words and neutral-valence words in RTs (472 vs. 468 ms, t(24) = 1.128, p>0.1) or in response accuracies (96.1% vs. 95.7%, t(24) = 0.499, p>0.1). The results of rating and questionnaire measurements from two subjects were missing because of technical problems. Analysis of the rating scores from the remaining subjects showed that subjects reported stronger negative emotional arousal to death-related words than to neutral-valence words (6.97±2.10 vs. 0.38±0.52; t(22) = 14.179, p<0.001).

#### ERP results

**Figure 1:**
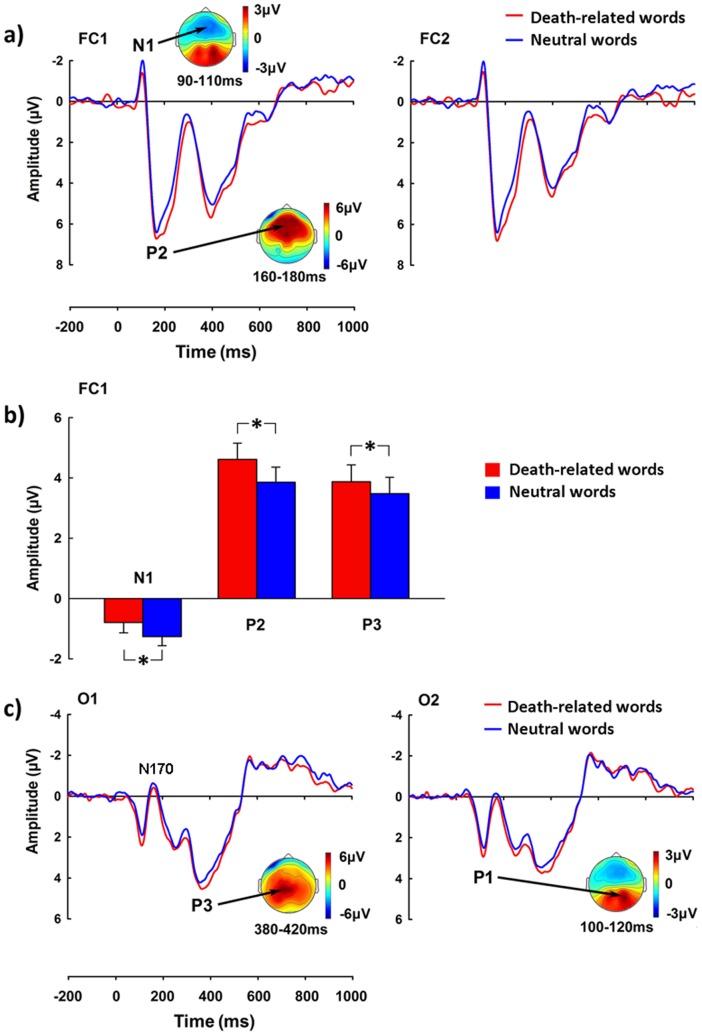
ERPs elicited by death-related words and neutral-valence words in Experiment 1. a) ERPs recorded at FC1 and FC2 and the voltage topographies of N1 and P2; b) The amplitudes of N1 (84–120 ms), the descending phase of P2 (160–300 ms), and P3 (300–500 ms). Error bars are standard errors; c) ERPs recorded at O1–O2 and the voltage topographies of P1 and parieto-occipital P3. *p<0.05.

Figure 1 illustrates grand-averaged ERPs to death-related and neutral-valence words and the voltage topography of each ERP wave. ANOVAs of the N1 amplitudes with Death Relevance and Hemisphere as independent variables showed a significant main effect of Death Relevance over the fronto-central and central electrodes (F(1, 24) = 4.723 to 7.928, p = 0.040 to 0.009), suggesting that the N1 was of smaller amplitude to death-related words than to neutral-valence words. The main effect of Hemisphere and its interaction with Death Relevance were not significant (ps >0.05). Death-related words also elicited larger amplitudes of the P1 over the parieto-occipital and occipital (F(1, 24) = 7.427 to 19.191, p = 0.012 to 0.0002) compared to neutral-valence words, whereas the main effect of Hemisphere and the interaction were not significant (ps >0.05). ANOVAs of the N170 amplitude did not show any significant effect (ps >0.05).

The initial examination of the ERP results suggested that word valence influenced the ascending and descending phase of the P2 amplitudes in a different fashion. Thus we analyzed the amplitude of the P2 wave separately for the ascending phase and the descending phase in all experiments. We found that that, relative to neutral-valence words, death-related words elicited a larger amplitude of the ascending phase of the P2 at 124–160 ms over the frontal, fronto-central, and central electrodes (F(1, 24) = 4.327 to 9.289, p = 0.048 to 0.006). The descending phase of the P2 at 160–300 ms was also of larger amplitudes to death-related than to neutral-valence words over these electrodes (F(1, 24) = 6.330 to 28.065, p = 0.019 to 0.00002). Neither the main effect of Hemisphere or nor its interaction with Death Relevance was significant (ps >0.05).

The P3 was of larger amplitudes to death-related than to neutral-valence words over the frontal, fronto-central and central electrodes (F(1, 24) = 4.304 to 7.456, p = 0.049 to 0.012). However, neither the main effect of Hemisphere or nor its interaction with Death Relevance was significant (ps >0.05). Death-related words also elicited larger P3 over the parietal, parieto-occipital, and occipital electrodes compared to neutral-valence words (F(1, 24) = 4.152 to 13.515, p = 0.053 to 0.001). A reliable main effect of Hemisphere was observed on the P3 amplitudes over the fronto-central (F(1, 24) = 4.905 to 18.952, p = 0.037 to 0.00006) and parietal electrodes(F(1, 24) = 8.784 to 13.710, p = 0.049 to 0.001), suggesting a larger P3 amplitude over the left than right hemisphere. However, there was no significant interaction between Death Relevance and Hemisphere (ps >0.05).

#### Correlation between ERPs and subjective ratings

**Figure 2:**
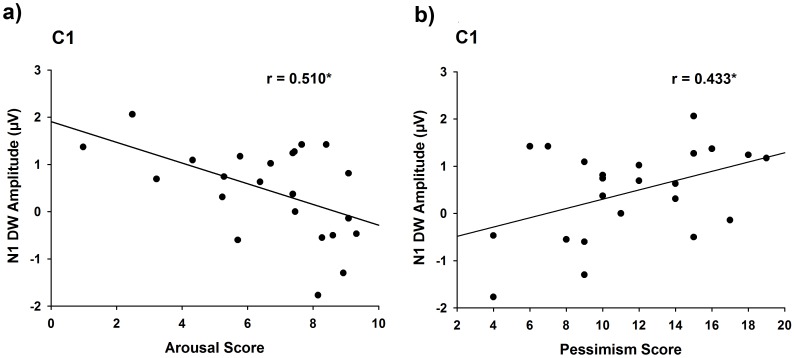
Correlation results in Experiment 1. (a) Correlation between the mean amplitudes of the difference wave at 84–120 ms recorded at C1/C2 and the differential negative emotion scores associated with death-related and neutral-valence words; (b) Correlation between the mean amplitudes of the difference wave at 84–120 ms recorded at C1/C2 and the pessimism scores across subjects. *p<0.05. DW = difference wave.

To investigate whether the neural activity underlying the processing of death-related words can predict subjective feelings associated with the stimuli, we calculated correlations between the amplitudes of difference waves (obtained by subtracting ERPs to death-related words from those to neutral-valence words) and differential rating scores associated with death-related and neutral-valence words. The amplitude of the difference wave in N1 time window over the fronto-central and central electrodes was negatively correlated with the differential emotional arousal rating scores (r = −0.430 to −0.586, p = 0.041 to 0.003, Figure 2a), the stronger arousal subjects felt about death-related words, the smaller amplitude of the N1 difference wave. Such correlations held even after controlling for the N1 amplitudes to neutral-valence words using partial correlation (r = −0.447 to −0.560, p = 0.037 to 0.007). Moreover, the N1 difference wave amplitude was positively correlated with the pessimism score in the LOT (r = 0.419 to 0.503, p = 0.047 to 0.015, Figure 2b), the more pessimistic an individual is, the greater the N1 modulation by death-related words. Such correlations also held even after controlling for the N1 amplitudes to neutral-valence words using partial correlation (r = 0.406 to 0.448, p = 0.061 to 0.037). These results suggest that correlations between N1 and subjective reports could not be due to the N1 fluctuation associated with neutral-valence words. However, the N1 effect did not correlate with subjective ratings of anxiety using DAS and EAQ (ps >0.05).

The ERP results of Experiment 1 revealed neurocognitive processes that differentiated between death-related and death-unrelated linguistic cues in a semantic unrelated perceptual task. Specifically, the early N1 amplitude decreased to death-related words compared to neutral-valence words. Interestingly, the N1 effect positively correlated with subjective reports of arousal and pessimism. However, the correlation between arousal and pessimism scores was not significant (p>0.1). Thus it appears that, although both arousal and pessimism scores correlated with the N1 effect, arousal and pessimism may be associated with different aspects of the processing of death-related words such as subjective feelings about death-related stimuli and attitude toward life. Similar to previous ERP studies of emotional Stroop effect using threat words [19], [20], we observed increased P2 to death-related words compared to neutral-valence words. The increased P3 amplitudes to death-related words suggest enhanced evaluative processes to categorize words in terms of death-relevance even when semantic meanings of the words were task irrelevant.

### Experiment 2

#### Behavioral performances

Paired-sample t-tests showed that neither RTs nor response accuracies differed significantly between life-related and life-unrelated words (465 vs. 463 ms, t(24) = 0.760, p>0.1; 96.8% vs. 95.9%, t(24) = 1.668, p>0.1). Subjects were not asked to rate to life-related and life-unrelated words after the EEG session because the rating scores of emotional arousal from the independent group of subjects were extremely low.

#### ERP results

**Figure 3:**
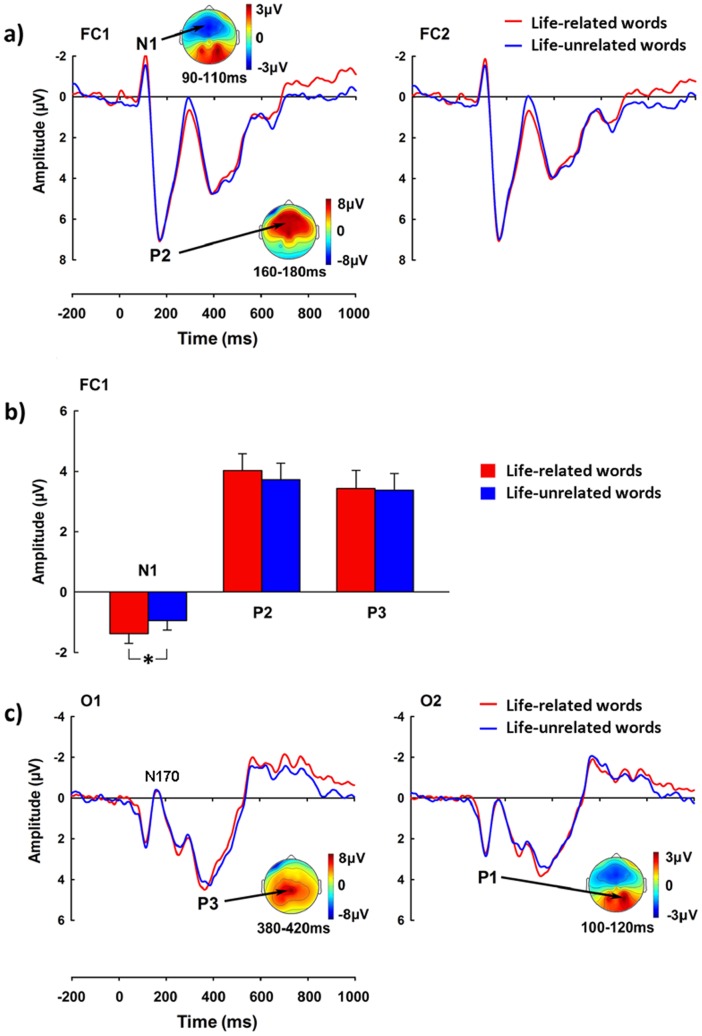
ERPs elicited by life-related words and life-unrelated words in Experiment 2. a) ERPs recorded at FC1–FC2 and the voltage topographies of N1 and P2; b) The amplitudes of N1 (84–120 ms), the descending phase of P2 (160–300 ms), and P3 (300–500 ms). Error bars are standard errors; c) ERPs recorded at O1–O2 and the voltage topographies of P1 and parieto-occipital P3. *p<0.05.

Figure 3 illustrates grand-averaged ERPs to life-related words and life-unrelated words and the voltage topographies of each ERP wave. Similar to those observed in Experiment 1, both life-related and life-unrelated words evoked a frontal N1 followed by the P2 and P3 waves, and an occipital P1 and N170. ANOVAs of the N1 amplitude with Life Relevance and Hemisphere as independent variables revealed a main effect of Life Relevance over the fronto-central and central electrodes (F(1, 24) = 4.433 to 5.046, p = 0.046 to 0.034). Neither the main effect of Hemisphere nor its interaction with Life Relevance was significant (ps >0.05). Analyses of the P1 and N170 amplitudes did not show any significant effect (ps >0.05). ANOVAs of the P2 amplitudes in the ascending and descending phases did not show any significant effect. Similarly, ANOVAs of the P3 amplitudes did not show any significant effect (ps >0.05).

#### Correlation between ERPs and subjective ratings

**Figure 4:**
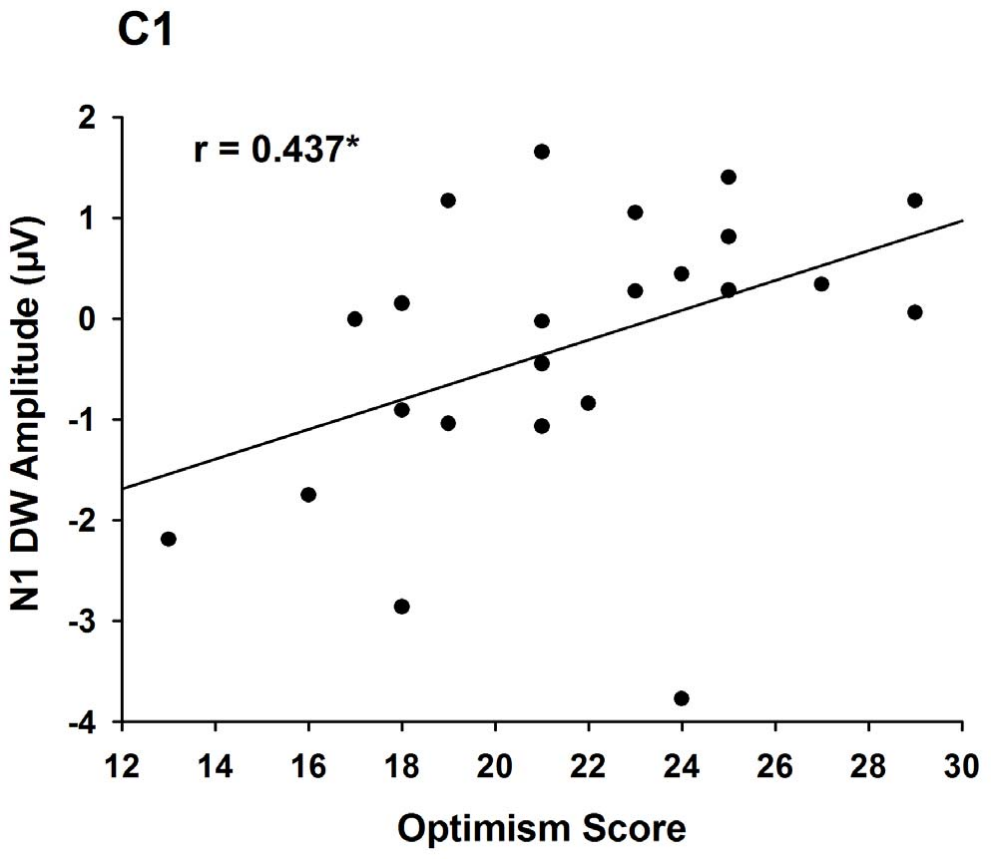
Correlation results in Experiment 2. Correlation between the mean amplitudes of the difference wave at 84–120 ms recorded at C1/C2 and the differential optimism scores. *p<0.05. DW = difference wave.

To examine if the neural activity underlying the processing of life-related words may predict subjects’ positive attitude toward life, we calculated correlations between the amplitudes of difference waves (obtained by subtracting ERPs to life-related words from those to life-unrelated words) and the LOT optimism rating scores. We found that the amplitude of the N1 difference wave over the fronto-central and central electrodes was positively correlated with the LOT optimism rating scores (r = 0.417 to 0.581, p = 0.048 to 0.004, Figure 4). Such correlations also held even after controlling for the N1 amplitudes to neutral-valence words using partial correlation (r = 0.425 to 0.557, p = 0.048 to 0.007).

Consistent with our hypothesis, life-related words induced larger N1 compared to life-unrelated words. Thus the early coding of life-related words is opposite to that observed with death-related words in Experiment 1. Such opposite neural coding of death-related and life-related words support the existence of early coding of life in linguistic cues. Interestingly, Experiment 2 did not find significant modulation of the onto-central P2 by life relevance. In comparison with the absence of the P2 effect in Experiment 2, one may hypothesize that the P2 results of Experiment 1 may reflect the processing of negative valence. If this hypothesis is correct, one may expect similar P2 effect when comparing negative-valence versus neutral-valence words as our previous study showed that subjects reported stronger negative emotion to both death-related and negative-valence words [11]. This was tested in Experiment 3.

### Experiment 3

#### Behavioral performances

There was no significant difference between negative-valence words and neutral-valence words in RTs (463 vs. 461 ms, t(24) = 0.552, p>0.1) and response accuracies (96.2% vs. 95.6%, t(24) = 1.141, p>0.1). Subjects rated negative-valence words with higher emotional arousal compared with neutral-valence words (4.71±1.83 vs. 0.38±0.52; t(22) = 10.409, p<0.001).

#### ERP results

**Figure 5:**
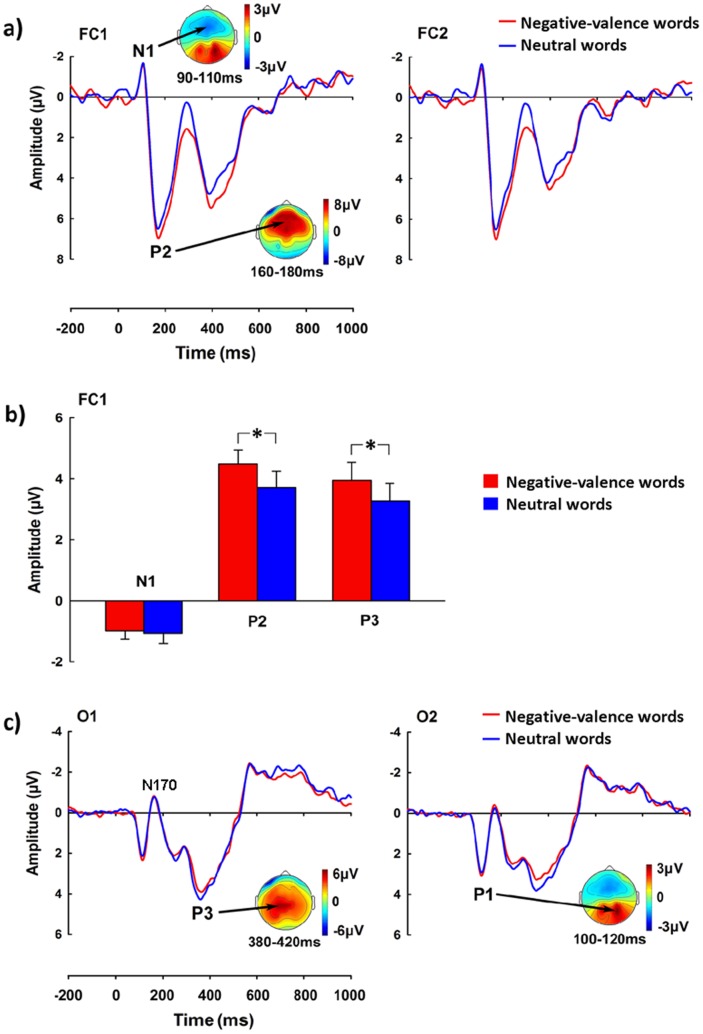
ERPs elicited by negative-valence words and neutral-valence words in Experiment 3. a) ERPs recorded at FC1-FC2 and the voltage topographies of N1 and P2; b) The amplitudes of N1 (84–120 ms), the descending phase of P2 (160–300 ms), and P3 (300–500 ms). Error bars are standard errors; c) ERPs recorded at O1–O2 and the voltage topographies of P1 and parieto-occipital P3. *p<0.05.

Figure 5 illustrates grand-averaged ERPs to negative-valence words and neutral-valence words and the voltage topographies of each ERP wave. Both negative-valence and neutral-valence words evoked a frontal N1 followed by the P2 and P3 waves. There were also occipital P1 and N170. ANOVAs of the N1 amplitude with Valence and Hemisphere as independent variables did not show any significant effect. Similarly, ANOVAs of the P1 and N170 amplitudes did not show any significant effect (ps >0.05).

While there was no significant effect of Valence on the amplitudes of the ascending phase of the P2 at 124–160 ms (ps >0.10), the descending phase of the P2 at 160–300 ms was of larger amplitudes to negative-valence than neutral-valence words over the frontal, fronto-central, and central area (F(1, 24) = 7.552 to 26.146, p = 0.011 to 0.00003). Neither the main effect of Hemisphere nor its interaction with Valence was significant (ps >0.05). ANOVAs of the P3 amplitudes showed a significant main effect of Valence at over the frontal and fronto-central electrodes (F(1, 24) = 4.871 to 10.756, p = 0.037 to 0.003), suggesting a larger P3 associated with negative-valence words compared to neutral-valence words. A reliable main effect of Hemisphere were also observed (F(1, 24) = 4.845 to 22.400, p = 0.038 to 0.00008), as the P3 was of larger amplitudes over the left than right hemisphere. However, there was no significant interaction between Valence and Hemisphere (ps >0.05). The P3 amplitudes over the parietal electrodes showed a significant main effect of Hemisphere (F(1, 24) = 10.41 to 15.85, p = 0.042 to 0.001), suggesting greater P3 amplitudes over the left than right hemisphere. However, neither the main effect of Valence not its interaction with Hemisphere was significant (ps >0.05).

We also conducted correlation analyses of subjective ratings of emotional arousal and the amplitudes of the difference wave but failed to find any significant results (ps >0.05).

The results of Experiment 3 first indicate that negative valence of words does not necessarily modulate the N1 amplitudes, providing further evidence that the N1 effect is specific for coding the negative side of life. In addition, Experiment 3 showed that negative valence of words resulted in modulations of the P2 amplitude, being enlarged to negative-valence words than to neutral-valence words. However, the P2 modulation was observed only at the descending phase of the P2 wave and occurred later than that observed Experiment 1. The results suggest that the processing of negative-valence of words describing negative events or actions may take place at a later stage compared with that of death-related words.

### Comparison Across Experiments

Separate analyses of the ERP amplitudes in Experiments 1–3 suggested differential neurocognitive processes of death-related, life-related, and negative-valence words. In particular, relative to that in the control condition, the N1 amplitude decreased to death-related words (Experiment 1), increased to life-related words (Experiment 2), but did not vary as a function of word valence (Experiment 3). To further confirm the different patterns of the N1 modulations across the three experiments, we first compared the N1 amplitude in the control condition across the three experiments. This did not show any significant difference (p>0.05), indicating the N1 amplitude was comparable in the three experiments. Next we normalized the N1 amplitudes by z-transforming the N1 amplitudes across the two conditions in each experiment. Finally, we conducted a 2 (Treatment vs. Control)×3 (Experiments 1, 2 or 3) ANOVA of the normalized N1 amplitudes over the fronto-central and central electrodes. The ANOVA showed significant interactions of Treatment×Experiment (F(2, 48) = 3.430 to 4.959, p = 0.041 to 0.011), indicating the different patterns of the N1 modulations across the three experiments. Post hoc analyses further confirmed that the normalized N1 amplitude was smaller to death-related words than to neutral-valence words in Experiment 1 (t(24) = 2.222 to 2.861, p = 0.009 to 0.036), was larger to life-related words than to life-unrelated words in Experiment 2 (t(24) = 2.569 to 3.229, p = 0.017 to 0.004), but did not differ between negative-valence words and neutral-valence words in Experiment 3 (ts <1.3, ps >0.1). Similar ANOVA of the normalized P2 and P3 amplitudes did not show any significant effect (ps >0.05).

In order to further confirm that the difference in N1 amplitude elicited by death-related and life-related words, we directly compared the N1 amplitudes elicited by death-related words and life-related words. ANOVAs with Lifehood (death-related vs. life-related) and Hemisphere (left vs. right) as independent variables revealed a significant main effect of Lifehood over the fronto-central and central electrodes (F(1, 24) = 4.40 to 5.65, p = 0.026 to 0.047), suggesting larger N1 amplitudes in response to life-related words than to death-related words. This effect was qualified by a significant interaction of Lifehood×Hemisphere (F(1, 24) = 4.23 to 9.03, p = 0.051 to 0.006). Post hoc analyses showed that modulations of the N1 amplitude by Lifehood was significant over the left hemisphere (t(24) = 2.21 to 2.85, p = 0.04 to 0.008) but not over the right hemisphere (ps >0.05). Taken together, these results suggest that the N1 wave may be involved in coding lifehood of linguistic cues and this process was dominated by the left hemisphere.

Finally, to test whether behavioral performances were different across the three experiments, RTs and response accuracies were also subjected to the 2 (Treatment vs. Control)×3 (Experiments 1, 2 or 3) ANOVA. Neither the main effect of Treatment nor its interaction with Experiment was significant (ps >0.1).

## General Discussion

In three experiments we recorded ERPs to death-related, life-related, negative-valence, and neutral-valence words in a modified Stroop task in order to clarify the neurocognitive processes of linguistic cues related to death. Although subjective ratings suggest discrepant negative emotional arousal linked to words from different categories, behavioral performances during EEG recordings did not differ between death-related (life-related or negative-valence) words and neutral-valence words, suggesting comparable task difficulty in naming colors of words in different categories. In addition, ERPs elicited by neutral-valence words in Experiments 1 and 3 did not differ significantly from those elicited by life-unrelated words in Experiment 2, indicating comparable neural activity in the control condition in the three experiments. Thus the difference in ERPs observed across the three experiments did not arise from the neural activity in the control conditions. Our ERP results highlight three stages of the online processing of linguistic cues of death.

First, death-related words decreased the N1 amplitude over the fronto-central and central area as early as 84 ms after stimulus onset compared to neutral-valence words. The N1 effect observed here cannot simply be attributed to negative emotional arousal for several reasons. First, the N1 effect occurred earlier than the neural modulations by threat words [19], [20] and those by negative-valence words [16]–[18]. The N1 effect was even earlier than the modulation of neural activity by emotional pictures which is characterized by enlarged negativity as early as 120 ms over the occipito-temporal area to pleasant compared to neutral and unpleasant pictures [33]. Second, the N1 effect observed in Experiment 1 was not observed for negative-valence words in Experiment 3 although subjects also reported greater emotional arousal to negative-valence compared to neutral-valence words. Moreover, the N1 modulation was evident for life-related and life-unrelated words that were rated low in arousal. Third, the arousal account would predict a larger N1 effect associated with greater arousal rating and a positive correlation between the N1 effect and the arousal rating. However, Experiment 1 found a negative correlation between the N1 effect and the arousal ratings. Taken together, the N1 modulation by death-related and neutral-valence words cannot simply be attributed to emotional arousal. The N1 modulation by death-related words indicates an early detection of linguistic cues prior to emotional responses elicited by death-related or negative-valence words.

Interestingly, modulations of the N1 amplitude by life-related words showed an opposite pattern compared to those by death-related words, i.e., the N1 amplitude was increased to life-related words but decreased to death-related compared to that in the control condition. The opposite N1 effects associated with death-related and life-related words are different from the ERP effects associated with negative and positive emotional stimuli. For example, pleasant and unpleasant faces elicited modulations of ERP amplitudes in the same direction (e.g., increased N230 over the frontal area) though pleasant and unpleasant faces are different in valence and arousal [34]. The N1 effect associated with life-related words cannot be explained by arousal because both life-related and life-unrelated words received low arousal ratings. The reverse N1 modulations by death-related and life-related words are consistent with the idea that ‘dead’ – the scarcity of life [15] – and ‘living’ are the two sides of the same coin (i.e., lifehood) and thus are possibly represented by the same early neural mechanism but in opposite patterns. Consistent with this, the N1 effect in Experiments 1 and 2 were respectively correlated with questionnaire measurements of opposite attitudes toward life (i.e., pessimism and optimism). However, the N1 effect did not correlated with self-reported death-related anxiety. The results suggest that individuals’ attitudes toward life rather than their general anxiety traits may influence the early stage of neurocognitive processes of death/life-related semantic cues.

The N1 effect associated with the processing of word categorization in terms of lifehood may reflect the neural representation of the self and related emotion. Both the N1 amplitude in the current work and the insular activity in our previous research [11] decreased to death-related words compared to neutral-valence words. We hypothesized that the decreased insular activity to death-related words possibly reflects weakened representations of the sentient self [11]. If the frontal N1 effect observed in Experiment 1 is associated with the weakened representation of the sentient self, it is then likely that the weakened representation of the sentient self might reduce negative emotional arousal, as suggested by the negative correlation between the frontal N1 effect and subjective feelings of negative emotion arousal linked to death-related words in Experiment 1. Interestingly, the decreased N1 amplitudes to death-related words compared to neutral-valence words positively correlated with the pessimism scores that reflect generalized expectancies concerning important future negative outcomes [35]. Individuals higher in pessimism tend to allocate more attention and maintain higher level of vigilance towards negative up-coming events [36], [37] and are thus possibly more sensitive to linguistic cues of death at the early stage of the processing stream. It should be noted that the N1 effect observed in our study does not mean that the N1 is only engaged in coding lifehood. Early ERP waves are also involved in perceptual and semantic processing of words [38]. The findings reported here extend our understanding of the functional role of the early neural activity in the processing of linguistic cues related to life and death.

The second stage of the processing of death-related words was associated with increased amplitudes of the frontal/central P2. The P2 effect was similarly observed with negative-valence words, though occurring slightly later, but was absent for life-related words. Thus the P2 effect was not specific to the processing of lifehood in linguistic cues but was associated with the processing of negative valence of words. This is consistent with previous findings that the P2 was enlarged by negative-arousing pictures [39], [40] and threat-related pictures or words [19], [20], [41]. As the P2 is associated with stimulus classification [42], the P2 effects observed in our work suggest that classification of words in terms of negative-valence may take place at a later stages of the processing stream compared to the initial categorization of words in terms of lifehood. Together, the N1 and P2 effects suggest that there is an early detection of linguistic cues of death which is followed by the processing of negative valence of words and induced emotional responses. The P2 effect associated with death-related words and the absence of the P2 effect associated with life-related words indicate that lifehood itself does not necessarily generate emotional responses. Only the negative side of lifehood (i.e., death) may automatically stimulate negative emotional responses.

The third stage of the processing of linguistic cues of death was characterized by the increased P3 amplitudes over the anterior and posterior scalp sites. The P3 effect was also observed with negative-valence words over the anterior scalp sites but was absent with life-related words, although the same color naming task was applied to these words. The P3 is believed to be engaged in stimulus evaluation at a late stage of cognitive processes [43], [44] and is augmented for emotional facial expressions relative to neutral expression [45], [46], [47]. Threat words also generated enlarged P3 relative to non-threat words in color naming tasks [19], [20], [48], [49]. Similar to our research, Klackl et al. [23] also found a late positive potential that was enlarged by death-related vs. death-unrelated words. Together, these findings suggest that death-related words might be more deeply evaluated compared to neutral-valence words and induce sustained motivated attention. However, this long-latency evaluation process was comparable for death-related words and negative-valence words describing events or actions with negative outcomes. In contrast, the color naming task did not lead to an enhanced evaluation of life-related words as these words do not have negative implications and do not induce early negative emotional response. Thus the neurocognitive process of linguistic cues related to death is also characterized by an evaluation process that is commonly observed with aversive stimuli in different domains. Previous findings suggest that there are two subcomponents of P3 [50]. The P3a originates from the frontal lobe and is engaged in stimulus-driven attention during task processing, whereas the P3b originates from temporal-parietal activity associated with attention and appears related to subsequent memory processing. In our study death-related words relative to neutral-valence words increased the P3 amplitudes over both the anterior and posterior scalp sites. It is likely that death-related words may enhance both attention and memory processing in the P3 time window relative to neutral-valence words. The P3 showed larger amplitudes over the left than right hemispheres regardless of word valence in our work. There is an inconsistent pattern of the P3 laterality in previous literatures of the Stroop effect. For example, Thomas et al. [20] found larger P3 over the left than right hemispheres in a word relevant task, whereas Franken et al. [47] observed larger P3 over the right than left hemispheres in a color naming task. It is currently unknown whether the differential P3 laterality observed in these studies arose from the difference in stimuli or task instructions, which can be assessed in future research.

One may notice that the occipital P1 wave was of larger amplitude to death-related words compared to neutral-valence words. The P1 effect was not observed with negative-valence and life-related words. However, the P1 modulation may not be limited to death-related words because previous studies also found enlarged P1 amplitudes to threat words relative to non-threat words [19], [20], [48], [49]. There has been ample evidence that the P1 modulation reflects attentional enhancements of visual extrastriate activity [51]. Thus the P1 effect suggest that both death-related and threat words might induce increased attentional bias in the early perceptual process, which may contribute to the late enhanced evaluative processes of stimuli.

Finally, while an increasing number of brain imaging findings uncover the neural substrates underlying death-related thought, a recent work also found brain imaging evidence that increasing mortality salience significantly modulates other cognitive/emotional processing. Luo et al. [52] scanned two subject groups when they viewed video clips showing others in pain after they were primed with death-related thought and negative affect in terms of fear/anxiety, respectively. It was found that perceiving painful vs. non-painful stimuli in the pre-priming session activated the midcingulate/dorsal medial prefrontal cortex, bilateral anterior insula/inferior frontal cortex, bilateral secondary somatosensory cortex, and left middle temporal gyrus. However, MCC/dMPFC activity in response to others’ suffering was decreased by the mortality salience priming but was not influenced by the negative affect priming. Moreover, subjective feelings of fear of death moderated the co-variation of MCC/dMPFC and left insular activity during perception of others in pain. These findings indicate that reminders of mortality may strongly affect other cognitive/affective processing in the human brain. This promising line of research may uncover the neural substrates underlying profound influence of death-related thought on human behavior.

In summary, our ERP findings highlight neural mechanisms underlying the processing of linguistic cues of death in three successive windows. These include an early detection of lifehood followed by encoding of negative valence, and a later enhanced evaluation of events and actions. The early detection of lifehood may be specific to linguistic cues of death, whereas the late valence encoding and evaluation are common for stimuli that are either aversive or implicate threat to survival. In addition, our results suggest that the early detection process of lifehood may be influenced by dispositional pessimism and affect subjective feeling of stimulus arousal. Our ERP findings expand our knowledge of human concerns about death.
